# How do Consumers Search for and Appraise Information on Medicines on the Internet? A Qualitative Study Using Focus Groups

**DOI:** 10.2196/jmir.5.4.e33

**Published:** 2003-12-19

**Authors:** Geraldine Peterson, Parisa Aslani, Kylie A Williams

**Affiliations:** ^1^Faculty of PharmacyThe University of SydneyAustralia

**Keywords:** Medicines, drugs, information, Internet, consumers, focus groups, qualitative research

## Abstract

**Background:**

Many consumers use the Internet to find information about their medicines. It is widely acknowledged that health information on the Internet is of variable quality and therefore the search and appraisal skills of consumers are important for selecting and assessing this information. The way consumers choose and evaluate information on medicines on the Internet is important because it has been shown that written information on medicines can influence consumer attitudes to and use of medicines.

**Objective:**

To explore consumer experiences in searching for and appraising Internet-based information on medicines.

**Methods:**

Six focus groups (N = 46 participants) were conducted in metropolitan Sydney, Australia from March to May 2003 with consumers who had used the Internet for information on medicines. Verbatim transcripts of the group discussions were analyzed using a grounded theory approach.

**Results:**

All participants reported using a search engine to find information on medicines. Choice of search engine was determined by factors such as the workplace or educational environments, or suggestions by family or friends. Some participants found information solely by typing the medicine name (drug or brand name) into the search engine, while others searched using broader terms. Search skills ranged widely from more-advanced (using quotation marks and phrases) to less-than-optimal (such as typing in questions and full sentences). Many participants selected information from the first page of search results by looking for keywords and descriptions in the search results, and by looking for the source of the information as apparent in the URL. Opinions on credible sources of information on medicines varied with some participants regarding information by pharmaceutical companies as the "official" information on a medicine, and others preferring what they considered to be impartial sources such as governments, organizations, and educational institutions. It was clear that although most participants were skeptical of trusting information on the Internet, they had not paid conscious attention to how they selected information on medicines. Despite this, it was evident that participants viewed the Internet as an important source for information on medicines.

**Conclusions:**

The results showed that there was a range of search and appraisal skills among participants, with many reporting a limited awareness of how they found and evaluated Internet-based information on medicines. Poor interpretation of written information on medicines has been shown to lead to anxiety and poor compliance to therapy. This issue is more important for Internet-based information since it is not subject to quality control and standardization as is written information on medicines. Therefore, there is a need for promoting consumer search and appraisal skills when using this information. Educating consumers in how to find and interpret Internet-based information on medicines may help them use their medicines in a safer and more-effective way.

## Introduction

Consumers frequently use the Internet as an information source and it has been reported that 80% of adult Internet-users have accessed it for general health information [1]. More specifically, 36% of Internet-using consumers have used the Internet as a source of information on medicines [1].

It is broadly acknowledged that health information on the Internet is of variable quality as evidenced by the large number of studies that have explored the quality of consumer health information on the Internet [2]. This is to be expected because the Internet is a free medium. It has also been widely postulated that consumers searching for health information are in danger of being harmed by poor-quality information even though there is little evidence of this [3]. A consumer's risk for encountering poor-quality health information is purportedly related to the proportion of poor-quality information on the Internet and the consumer's ability to filter out this information [2]. As the quality of information on the Internet cannot be controlled, the more-imperative issue is the ability of consumers to search through information and assess its quality so they are able to avoid untrustworthy information [4]. An Australian study suggested that consumers found it difficult to describe how they distinguished good-quality information on medicines from poor-quality information on medicines on the Internet [5]. However, this study was limited by a small (N = 9), select sample and did not explore in-depth the way consumers searched for and selected information on medicines.

There is little information concerning consumer Internet-search behavior for health information. One study reported that participants mainly select Web sites that looked and read professionally and preferred understandable Web sites from official sources that used scientific references [6]. When participants were observed while searching for health information on the Internet, it was found that they mainly used search engines and were described as having "suboptimal" search skills [6]. This study reported that participants did not find blatantly-incorrect health information in their searches [6]. This indicates that they had used selection criteria to decide on the Web sites, though the criteria were not fully described in this paper.

Consumer use of information on medicines is an important issue because written information on medicines has been shown to influence consumer attitudes towards their medicines, and affect their medicine-taking behavior [7]. Furthermore, medicines, unlike general health issues, have overtly-commercial imperatives, which may influence the information available. Since the Internet has become a common source of information on medicines, it is important to identify the way consumers are using it. Therefore the aim of this study was to explore consumer use of Internet-based information on medicines. In particular, the objectives were to:

- examine consumer attitudes to the availability and quality of Internet-based information on medicines;
- explore consumer reasons for using this information;
- explore consumer experiences in searching for and appraising information on medicines;
- investigate the self-reported impact and application of this information.

This paper will present results from the broader study on consumer experiences in searching for and appraising Internet-based information on medicines.

## Methods

### Selection of Method

Focus groups were selected to address the study aims because they are useful for time-efficient, in-depth exploration of issues surrounding topics where there is little information [8- 10]. Since there is little known about how consumers use Internet-based information on medicines, focus groups were an ideal method for exploring this issue. The results of focus groups are not intended to be statistically generalizable, but are used to reveal the range of consumer opinions and attitudes.

### Research Instrument

An interview guide consisting of general themes constructed from the literature was prepared (Table 1). This paper focuses on results ensuing from the exploration of themes 4, 5, and 6. The interview guide was composed of open-ended questions that addressed various issues pertaining to consumer use of Internet-based information on medicines; the questioning route was designed to stimulate discussion [11- 13].

**Table 1:** Themes for focus group interview guide*

General opinions about the Internet as a source of information on medicines. Experiences in using the Internet to seek information on medicines. Reasons for seeking information on medicines. The methods and process of searching for information on medicines. Opinions and critique of the information found. Experiences in the evaluation of the quality of Internet-based information on medicines. Feelings after reading the information. Actions taken as a result of reading the information. Perceived benefits and drawbacks of the Internet as a source of information on medicines

^*^ This paper focuses on results ensuing from the exploration of themes 4, 5, and 6.

The interview guide and questioning route was pretested with a convenience sample of consumers (N = 13) to test for interpretation, appropriateness, and comprehensiveness, and to establish face and content validity. No significant changes were made to the interview guide or questioning route as a consequence of this pretest.

### Participant Recruitment

After approval was granted by the Human Research Ethics Committee of The University of Sydney, participants were enlisted for the focus groups by a recruitment agency. Participants were recruited from the agency's database of consumers across metropolitan Sydney, Australia via telephone using a screening questionnaire.

Consumers were deemed to be eligible for this study if they had sought Internet-based information on medicines in the preceding 12 months. This bounded reference period was applied to allow for a suitable recall of past events [14] while also allowing enough time for consumers to have used the Internet for this purpose. Inclusion criteria required that participants were 18 years of age or over, did not require a translator to take part in focus group discussions, did not have training as a health professional, and did not have specialist Internet training. Participants were financially reimbursed for their time and travel expenses.

### Study Design

Six focus groups were conducted in a number of locations around metropolitan Sydney in March to May 2003.

To approximate a representative cross section of consumers, participants were recruited with the intention of including subjects from both genders and across different age groups. Focus groups were age stratified to achieve a level of homogeneity within each group. The use of stratification may increase congruency between participants, thereby allowing a more comfortable discussion [11,15]. Eight persons were recruited for each focus group to ensure that groups were large enough to motivate a discussion, yet small enough allow for all opinions to be heard [11]. The number of groups needed was not determined beforehand because data was collected until saturation occurred (the point where no new themes emerged) [10]. In this study, saturation occurred by the sixth focus group.

The focus groups were facilitated by a skilled moderator while 2 assistant moderators observed and took notes. The group discussions were 1 to 1.5 hours in duration and were digitally sound recorded after permission was obtained from all participants. The recordings were transcribed verbatim. Participants also completed a demographics questionnaire that collected data on Internet usage.

### Data Analysis

The verbatim transcripts were entered into NVivo qualitative software [16] and thematically content analyzed using a grounded theory approach. The grounded theory approach is an inductive approach to analyzing qualitative data, where ideas and emerging themes are systematically coded to generate theory [17].

## Results

This paper presents participants' responses to themes 4, 5, and 6 (Table 1). Responses to other themes are currently unpublished.

### Demographics

Forty-six consumers participated in this study. The age of the participants ranged from 18 to 67 years, with a median of 41 years (interquartile range, 21 years) and a mean of 41.7 years (standard deviation, 12.7 years). Fifty-seven percent of the participants were female. The majority of the participants were employed full-time (58.7%) and about a fifth were either retired or full-time homemakers. Almost half the sample (47.8%) had occupations that could be classified as managers, professionals, or associate professionals [18]. A high proportion of the sample (65.2%) had completed further educational qualifications beyond high school, and 23.9% of the sample had a bachelors or postgraduate degree.

Data on participant usage of the Internet is presented in Table 2. The majority of participants had a few years experience in using the Internet and over half had accessed it from both their home and workplace. In addition to using the Internet for information on medicines, most participants also used it for general health information and for services such as e-mail.

Data on participant usage of the Internet for information on medicines is presented in Table 3. In addition to using the Internet, many participants also reported using other media such as magazines for information on medicines. This variety of information sources has also been seen in another Australian study on consumer use of Internet-based general health information [19]. Even though most participants (82.6%) were seeking information for themselves, many reported also searching for other family members. This was also reflected in the aforementioned Australian study that showed that 63% of Internet-using consumers sought health information mainly for themselves [19].

**Table 3:** Participant usage of the Internet for information on medicines (N = 46 participants)

**Characteristic**	**Usage**	**Frequency, Number of Participants**	**Relative Frequency, (% of Participants)**
Media sources of information on medicines (more than one category could be selected)	Internet Magazines Television Books Radio	46 31 23 22 9	100.0 67.4 50.0 47.8 19.6
Person that Internet medicine information was used for (more than one category could be selected)	Self Spouse/partner Child Parent Another relative Friend	38 24 19 17 13 6	82.6 52.2 41.3 37.0 28.3 13.0
Health categories for which information on medicines had been sought for (more than one category could be selected)	Allergies Arthritis/joint pain Asthma Cancer Skin disorders Hormones Other miscellaneous Child health Diabetes High cholesterol Immunization Pain and injury High blood pressure Mental health Digestion/stomach disorders Infections Migraine Osteoporosis Alzheimer's disease Dementia	20 14 14 13 12 11 11 10 10 9 9 9 8 8 6 6 6 6 5 5	43.5 30.4 30.4 28.3 26.1 23.9 23.9 21.7 21.7 19.6 19.6 19.6 17.4 17.4 13.0 13.0 13.0 13.0 10.9 10.9

**Table 2:** Participant usage of the Internet (N = 46 participants)

**Characteristic**	**Usage**	**Frequency, Number of Participants**	**Relative Frequency, % of Participants**
Length of experience in the use of the Internet	More than 5 years 4 to less than 5 years 3 to less than 4 years 2 to less than 3 years 1 to less than 2 years Less than 1 year	17 13 7 4 4 1	37.0 28.3 15.2 8.7 8.7 2.2
Location of Internet access	Home and work Home only Work only	25 16 5	54.3 34.8 10.9
Activities that the Internet is used for (more than one category could be selected)	Information on medicines E-mail Health information Travel information/booking Banking/financial services News, weather, sport Job or study related research Real estate Shopping—product research Games and hobbies Chat or instant messaging Shopping—purchasing Purchasing medicines	46 45 43 40 36 34 34 33 33 26 22 19 7	100.0 97.8 93.5 87.0 78.3 73.9 73.9 71.7 71.7 56.5 47.8 41.3 15.2

### Searching for Internet-Based Information on Medicines

#### Search Engines

All participants had used a search engine to find information on medicines. Most participants had a single favorite search engine that they would always use, but a few reported using more than one search engine to find the information they required.

The choice of search engines was determined by many different factors ranging from the default search engine on their browser to active selection based on self-developed criteria. Numerous participants were influenced by the search engine that was used by coworkers, for example:

Group 4, Participant 8 I saw it on this guy's computer and . . . I thought 'Oh, I'm going to use this'. That's how I started it at work.

Some participants also reported that their browser automatically defaulted to a certain search engine and a few participants were unable to identify the search engine they used, for example:

Group 3, Participant 6 Couldn't tell you [the search engine] really. I just log on and use whatever comes on.

Many participants used search engines recommended by family and friends.

There were certain determinants that led some participants to actively choose a specific search engine. These included perceptions of the credibility of the search engine, ease of use, relation with services such as e-mail, and a lack of advertising. These determinants did not necessarily include perceived quality of the information on medicines obtained through their use.

A few participants reported using AltaVista [20] because they thought it had an educational advantage, for example:

Group 1, Participant 1 It's got an educational edge, that's my experience. When I was at university doing my second degree, that was one that was sort of promoted as credible I suppose.

Some participants preferred to use Ask Jeeves [21] because they could enter the searches in a question or statement format rather than using search terms.

Many participants reported using Yahoo! [22] because it appeared as a default homepage, was used as a personal e-mail account, or was advertised through other media. Yahoo! and Google [23] were also said to be useful for Australian-only searches.

Google was undoubtedly the search engine the majority of participants used most and preferred. This was especially true of the younger participants. The common perception was that Google appeared to be straightforward and did not focus on advertising, for example:

Group 4, Participant 6 It's just got less [rubbish]. It seems to be direct to what you want. I think that other [search engines] always have these categories and they always have suggestions for buying things and stuff like that but Google's pretty much straight to the point. It's simple.

Participants also commented that this search engine was useful for suggesting spelling corrections when errors were made, as medicine names were sometimes difficult to spell. A few participants reported preferring Google as their search engine of choice specifically for health-related searches but were unable to explain reasons for their preference.

Other search engines used by participants were metasearch engine Dogpile [24], Australian metasearch engine Search66 [25], Australian-based search engine Web Wombat [26], and ninemsn [27], the Australian-based access to search engine MSN Search [28]. Many participants who used metasearch engines were unaware of the difference between these and normal search engines.

Generally, although a variety of search engines were used by participants when seeking information on medicines, the majority of participants used the same few dominant search engines. Participants generally preferred search engines with less advertising, and would continue to use the same search engine if they were successful in their searches. Most participants used the same search engine that they used for nonhealth information, and were usually influenced by what was used by friends, family, and colleagues.

#### Search Processes

Participants displayed a large variation in the process of searching for information on medicines.

Most participants found information by typing the name of the medicine (drug name or brand name) into the search engine. A few participants felt this was the only way of finding information on a medicine, for example:

Interviewer How do you put in your searches?

Group 6, Participant 2 Medicines are really specific to just the name.

Other participants reported looking for broader information, for example:

Group 1, Participant 2 I often use a more general [search]. I might use something like 'women's health' or something. And I like to see a whole range of things . . . rather than targeting specifically . . . and then I choose within that.

The information found through this type of search was said to be less specific to one medicine and had more general or comparative information.

Some participants used more-advanced search techniques such as quotation marks, phrases, and extra words to narrow down their searches. They displayed an understanding of how these techniques helped to focus their searches, for example:

Group 4, Participant 6 If you type it in with quotation marks, it'll search for those words together whereas if you type them separately, it'll just search for them anywhere.

Participants reporting advanced skills were generally observed to be those who were younger or those who had greater experience of the Internet through work or study.

However, it was clear that search skills varied significantly. The following interchange illustrates the mixed levels of understanding as to how search engines work:

Group 5, Participant 4 [You need to] ask a specific question . . . 'What are the side effects?' rather than typing in 'penicillin'.

Participant 6 Yeah, you really have to do a whole sentence. A whole statement.

Participant 3 I would type in 'penicillin side effects'.

Participant 4 'Then it could hit on 'penicillin' or it could hit on 'side effects'.

The uninformed way in which some participants agreed upon what they considered to be optimal search skills was obvious in the group discussions. The majority of participants in this study who reported searching using less-than-optimal techniques—such as typing in whole questions—tended to be nonworkers, for example, full-time homemakers or retirees.

The search skills of participants varied widely and these differences may affect the resulting information that participants encounter. Searching via a search engine however, was not the only way of finding information on the Internet on medicines.

#### Other Methods of Finding Internet-Based Information on Medicines

Some participants mentioned ways of finding information on medicines in addition to using search engines.

A few participants said that they guessed the Web sites of medicines by typing the name of the medicine in the address bar in the format of www.[brand name or drug name].com.

Several participants found information on medicines from Web sites recommended by family and friends, and from seeing advertisements in seniors' and health publications. Some reported bookmarking favorite Web sites for future reference and a few subscribed to mailing lists at health-related Web sites.

One participant described searching for information on medicines using online journals. Although aware that the information was not aimed at consumers, this participant still chose to use this means to search for pertinent information on medicines:

Group 2, Participant 2 I actually searched via . . . the professional journals . . . And I guess that was a little bit harder to do it that way because . . . reading through the journals was quite difficult. I tend to just go to the abstracts.

Participants reported using a variety of search skills to obtain information on medicines. However, the important issue was how they selected and appraised the information.

### Appraising Internet-Based Information on Medicines

#### Selecting Internet-based Information on Medicines

Participants described different ways of choosing which Web site to visit when selecting from the numerous results obtained from using a search engine. Some worked down the list of results from the first one while others looked for keywords in the Web site descriptions or for the Web site's recency. Often participants made a judgment based on the URL (Web page address) of the result, for example:

Group 4, Participant 1 I actually like looking at the actual web address, just seeing how professional it is. Like if it's some silly thing, I won't bother going into it.

Many participants also reported looking for indicators in the Web site address to determine whether it belonged to a government, a university, an official organization, or a pharmaceutical company.

Even though most participants said they would not go beyond the first page of the search results, one expressed the opinion that the best information was in the middle of the results and not on the first few pages. This participant had the erroneous opinion that the first pages of results are older and that results appeared mainly in the order in which the information had been created.

Many participants reported looking for the country of origin of the information and preferred information generated from their country of residence, for example:

Group 4, Participant 2 If I'm searching for a medication . . . and it brought up some things and I noticed it was in Australia, I click on that.

These participants felt that Australian information would be more applicable to them and professed an awareness of health-setting issues such as differences in the brand names and availability of medicines in different countries. However, others had more confidence in United States-based information because they believed that this was where most new research was undertaken.

It was clear that most participants did not pay conscious attention to how they selected Internet-based information on medicines, with one referring to the process as "a vibe" that you obtain through experience. Another described this as a feeling that "things have a look of credibility." Similarly, many participants had trouble in articulating their selection process, for example:

Group 5, Participant 3 I find that sometimes I get to a site and I think 'Gee, this is a good site, but I don't know how I got there.' You know what I mean? You fluke it.

Despite the inability of many participants to express how they selected information on medicines, many were able to express what they would not select. Participants reported quickly rejecting sites that were slow to load, sites that contained too many graphics, and sites that had pop-up advertisements.

The process of selecting information on medicines varied among the participants. It appeared that all participants had their own criteria for selecting and rejecting information which may or may not appear logical to others. Credibility of the source, however, appeared to be a common determinant in the criteria of all participants.

#### Credibility of the Source of Internet-Based Information on Medicines

Participants expressed conflicting opinions about the credibility of the source of Internet-based information on medicines. Many participants regarded information produced by pharmaceutical companies to be the "official" information on a medicine and therefore trusted this the most, while many others were suspicious of a possible information bias, for example:

Group 1, Participant 7 If you're looking at [a pharmaceutical company website], they've got factories throughout the world, they're a pretty good company so . . . you know that they've done so much research it's credible information.

Group 1, Participant 6 If it's a pharmaceutical company, they're gonna put a good stance on their drug.

Many other participants preferred information that originated from what they considered to be impartial and reputable sources such as government, professional, or disease-focused organizations, or university Web sites. A few participants also reported looking for credentials such as the author's qualifications when assessing the credibility of the information provider.

A small number of participants preferred information written by other consumers who had personal experiences in taking the medicine. However, most participants expressed that they would be less likely to trust information on medicines generated by other consumers, for example:

Group 1, Participant 6 There are chat rooms . . . if you've ever been prescribed such and such a medication; you'll get people from all around the world . . .

Participant 2 Do you not find that a bit dangerous because everything is rather specific to each person's body?

Participant 6 Oh yeah, but it would be comparable to having a chat with some of your friends.

Some participants felt that the authorship of Internet-based information on medicines should be regulated and feared the reliability of the information because there was "no watchdog" for the information published on the Internet while others regarded it as analogous to the way they would trust information given in common conversation and therefore felt comfortable using information in this context.

The credibility of the source of information on medicines was a strong determinant in the selection process. However, in addition to the source participants evaluated information using criteria described in the next section.

#### Evaluating Internet-Based Information on Medicines

Participants evaluated information on medicines using criteria such as the motive for the information, the language used, and the applicability to their needs.

Almost all participants were skeptical to some degree of Internet-based information on medicines. Many participants professed a universal need for consumers to inherently distrust this information, and to interpret it accordingly. One participant stated that it is important to also consider why the information is on the Internet:

Group 1, Participant 6 What are the motives? Are they conflicting, credible? Whoever has posted it, are they trying to make a profit?

Other participants described the obviously difficult-to-believe nature of some of this information and looked for signs of conspiratorial or misleading language when deciding whether to trust the information, for example:

Group 2, Participant 8 If it says 'hazard free' and 'completely no side effects', for example, I'm more likely to disbelieve than believe that

In addition to this awareness of unreliable information on medicines, many participants also expressed an understanding that the information they find may not necessarily be applicable to them and that the information should not be used at face value, for example:

Group 2, Participant 7 The thing with medicines is there's no sort of right or wrong . . . Everyone's different, everyone's going to have a different reaction.

Group 2, Participant 6 When you ask the doctor, they tell you 'well, [the side effects] happen but it's not like that', I think what happens is that the information is not tailored for myself. It's general information.

Pertinent to this appraisal was the information-filtering process described by participants:

Group 2, Participant 4 It's always better to try and take as much information and try and sift out what's useless

Group 1, Participant 6 When they're talking about people using this medicine, 'ninety-eight percent will die within five years' . . . you have to take that and filter it through a whole bunch of other variables . . . and whether [the information] is not terribly well informed or completely informed.

One common way in which some participants were able to filter information on medicines was to use other Web sites for comparison and cross-checking, for example:

Group 4, Participant 1 I always go to two or three sites.

Although participants reported methods of evaluating information, many expressed a difficulty in their evaluation, for example:

Group 2, Participant 4 How do you [figure] out what's useful?

Group 3, Participant 7 How do you know what's reliable and what's not?

Ultimately, despite an awareness of the shortcomings and difficulties in evaluating the quality of information on medicines, all participants saw the Internet as an important resource for this information, for example:

Group 1, Participant 1 I think as patients you expect immediate information and the Internet, whether it's credible or not, it's the fact that people can get it.

## Discussion

The issue of consumer use of Internet-based information on medicines is important because it has been shown that written information on medicines can be interpreted by consumers in ways that may lead to anxiety or apprehension [7,29- 32], and a refusal of prescribed medicines [33]. Conversely, it has been shown that written medicine information increases consumer knowledge about their medicines [29,34- 36] and that well-informed consumers with an increased understanding of the purpose of their medicines may have improved compliance and satisfaction with their therapy [29,31,37- 40].

However, studies on consumer use of written information on medicines have evaluated standardized information on medicines such as that produced by pharmaceutical companies, government or professional bodies, or health care practitioners [7]. In contrast, this study explored Internet-based information, which is neither standardized nor subject to universal quality control. Furthermore, medicines in particular are subject to commercial considerations that may have an impact on the motives for and quality of information. Therefore, the impact of Internet-based information on consumer use of medicines may differ from that reported from consumer use of standardized written information on medicines.

The reported search skills of these participants were comparable to those of participants observed while searching for general health information [6] in that they mainly searched using simple strategies in a search engine and chose results primarily from the first page of search results. Although this similarity is not surprising, it does illustrate the overlap between appraising general health information and specifically medicines-related information. Indeed, it was not always possible for consumers in this study to speak on issues surrounding searching for and appraising information on medicines without speaking about other health-related issues.

Participants in this study searched for information on medicines using a range of search techniques from simple 1-word searches and advanced techniques to suboptimal techniques. However, although some participants had little understanding of how search engines worked and possessed suboptimal search skills, a few participants described proficient search skills. Contrary to findings where consumers were observed to use information not applicable to their health setting [6], participants generally reported a strong awareness of the limitations of non-Australian information due to health-setting limitations pertinent to medicines use.

Participants were conscious that there was an abundance of poor-quality information on medicines on the Internet. They were also predominantly aware that information on the use of medicines and on the incidence of side effects is often based on individual factors that should not be seen as applicable to everyone. Therefore, while consumer evaluation skills have been referred to as "meager" [41], the assumption that consumers believe everything they read does not take into account those participants who are savvy about issues such as bias, commercialism, and the lack of regulation of Internet-based information on medicines.

However, the fact that many participants searched for information on a medicine by typing the brand name into a search engine would indicate that it was highly likely that they encountered the Web site of a pharmaceutical company on the first page of results [42], which raises the matter of consumer ability to interpret information on medicines that may not be comparative and unbiased in nature and not aimed at an Australian audience. Even though results from this study would indicate that many participants were aware of these limitations, others still viewed a pharmaceutical company Web site as the official, and therefore exclusive, information on a medicine; this indicates that some consumers may be unaware of or uninterested in information on medicines produced by alternate sources. Nevertheless, it has been suggested that consumers are more likely to search for alternate sources, rather than relying on product brands, as they become more experienced using the Internet [43].

It is clear that there was a variety of skills among participants. Many had not been conscious of some of the issues surrounding the process of searching for and appraising information on medicines and did not undertake this process in the most-constructive way. Furthermore, there have been few studies in the literature that have sought to educate consumers on strategies for effective use of the Internet for health information [44- 47].

### Limitations in This Study

There are several important limitations in this study.

First, as this information is self-reported, consumers may not actually search for and appraise information in the same way as they describe. Such a discrepancy was demonstrated when participants in an observational study were reported to be less likely to look for the sources of the information than was apparent from claims in focus groups [6]. However, participants in that observational study were not searching for information that they would personally use; this may have meant that they were less concerned about the quality of the information.

Second, the bounded period of 12 months in the inclusion criteria may be too long for consumers to correctly remember details of how they searched for and chose information. It might have been beneficial to actually perform a search as an activity to stimulate the participants' memories.

Third, participants in group situations may feel compelled to provide socially-desirable answers that are not necessarily accurate. In this study, we sought to minimize this by informing participants that their results would be confidential and that they were welcome to speak about anything they felt even if they disagreed with someone else. However, this does not negate the problem. Although the use of individual interviews may help to minimize this discrepancy, this method is more time-consuming and cannot use group interaction for the generation of ideas.

Last, certain actions are intuitive and therefore difficult to articulate. Most participants were not able to adequately describe their search and appraisal processes, which suggests that this process may largely be a form of tacit or implied knowledge.

Therefore, future research needs to take into account actual observed (rather than reported) search and appraisal skills of consumers who are seeking information on medicines for their own use.

### Conclusion and Future Research

The results of this study show that consumers may benefit from greater awareness and education on the significance of good search and appraisal skills for information on medicines so that this process is deliberate and conducted with thought rather than being random and tacit. Furthermore, there is evidence that consumers may support education that shows them how to search for information on medicines on the Internet [48]. However, health promotion and education needs to take into account the variety of consumer skills in both searching for and critically evaluating information. Pharmacists are in an ideal position to provide consumer training as they frequently counsel consumers on medicines [49] and have consumers present them with information from the Internet [19]. However, to successfully deliver this program, pharmacists need to be trained in these skills . Furthermore, the impact of pharmacist education on consumers' searches for Internet-based information on medicines and appraisal of that information needs to be evaluated. Therefore, future research by this team will be on the development of a health-promotion program for pharmacists to train consumers to search for and appraise Internet-based information on medicines.
